# Far-UVC Radiation for Disinfecting Hands or Gloves?

**DOI:** 10.3390/pathogens12020213

**Published:** 2023-01-29

**Authors:** Martin Hessling, Ben Sicks, Bernhard Lau

**Affiliations:** Institute of Medical Engineering and Mechatronics, Ulm University of Applied Sciences, Albert-Einstein-Allee 55, D-89081 Ulm, Germany

**Keywords:** hand hygiene, far-UVC radiation, 222 nm excimer lamp, gloves

## Abstract

(1) Background: Far-UVC radiation in the spectral range 200–230 nm has, according to previous findings, a strong antimicrobial effect on pathogens, but exhibits hardly any harmful effect on human skin. Therefore, the present study will discuss whether such radiation could also be suitable for hand disinfection in the healthcare sector. (2) Methods: Hands and gloves were microbially contaminated and exposed to radiation from a 222 nm krypton-chloride-excimer lamp. The applied doses were 23 mJ/cm^2^ and 100 mJ/cm^2^, respectively. Irradiated and non-irradiated hands and gloves were pressed onto agar plates and colonies were counted and compared after 24 h of incubation. For comparison, we also treated hands and gloves with a commercial liquid alcohol-based disinfectant. (3) Results: On the hand, the 23 mJ/cm^2^ resulted in the reduction of the observed colonies on the agar plates by one log level. For the gloves irradiated with 100 mJ/cm^2^, a colony reduction of 1.3 log levels was recorded. In the comparative experiments with the commercial disinfectant, a colony reduction of 1.9 and approximately one log level was observed on hand and gloves, respectively. (4) Conclusion: In both cases, far-UVC radiation provided a considerable reduction in microorganisms. However, compared to published far-UVC irradiation results in suspensions, the disinfection success on hands and gloves was rather low. With regard to the irradiation limits currently existing in the European Union, multiple daily hand disinfection with far-UVC radiation is actually legally not possible at present, but the thresholds are currently under discussion and could change in the future. Far-UVC disinfection of hands in gloves seems theoretically possible if attention is paid to potential perforations in the gloves.

## 1. Introduction

Hand disinfection is one of the most important measures to reduce the spread of nosocomial infections in hospitals [1,2,3]. The standard is to rub the hands for a typical duration of about 30 s to a few minutes [1,4,5] with liquid, mostly alcohol-based disinfectants [1,2,3].

This hand disinfection procedure is usually carried out many times a day by healthcare workers, and this frequent application can lead to skin irritation and eczema, which is one of the reasons why compliance with hand disinfection is only around 40–50%, with a high variability [1,2,3]. Another disadvantage of hand hygiene with widespread alcohol-based disinfectants is that they work well against bacteria, fungi, and viruses, but only poorly against bacterial spores or protozoa [1].

Other widely used disinfection techniques, such as the application of heat or antimicrobial ultraviolet radiation, previously seemed very far-fetched, as these usually also lead to the damage of human tissue. However, almost 20 years ago, Sosnin et al. [6] found that there is short-wavelength UVC radiation, which damages eukaryotic cells less than prokaryotic cells. Meanwhile, it has been found that human skin tolerates UV radiation in the so-called far-UVC range of about 200–230 nm relatively well [7,8,9,10,11,12,13]. This radiation is almost completely absorbed in the stratum corneum and does not reach deeper skin layers. Nevertheless, the antimicrobial effect of this radiation on bacteria and other pathogens, including bacterial spores and protozoa, is similar to the known impact of germicidal 254 nm radiation of mercury vapor lamps [14].

Currently, the most common far-UVC sources are krypton-chloride-excimer lamps. These have a strong emission peak at a wavelength of 222 nm. Potential longer wavelength emissions around 260 nm, which could lead to DNA damage, are usually blocked by filters [15,16].

However, people must not be indefinitely exposed to this 222 nm radiation despite its seemingly low hazard potential. In Germany and the EU (European Union), there are limits for the repeated irradiation of human skin, which for 222 nm is currently just below 23 mJ/cm^2^ per day [17], though these limits are under discussion—at least in Germany [18]. In the United States, the ACGIH (American Conference of Governmental Industrial Hygienists) rose the daily threshold to just below 478 mJ/cm^2^ in early 2022 [19], based on recent studies of the effects of far-UVC on skin. It should be mentioned that the ACGIH limit for eyes is lower, with a maximum 222 nm dose of about 160 mJ/cm^2^ per day, and therefore the eyes might have to be protected if there is a danger of over-exposure.

The log-reduction doses of most pathogens are well below these threshold values. For example, *Staphylococcus aureus, Pseudomonas aeruginosa*, and severe acute respiratory syndrome coronavirus type 2 (SARS-CoV-2) in solution require 222 nm irradiation doses of 3.4, 2.0, and 1.2 mJ/cm^2^, respectively, for 90% reduction [20,21].

These log-reduction doses are clearly or very clearly below the above-mentioned limit values of 23 and 478 mJ/cm^2^, respectively. This raises the question of whether this radiation could also be applied for hand disinfection. For this purpose, it should not only be as harmless as possible to the skin, but also actually achieve a bacterial reduction on the skin. Despite the above results in solutions, this is not clear, as the skin has pores and folds that partially protect microorganisms against the radiation.

Therefore, in the small study presented here, the maximum daily irradiation dose currently permitted in Germany will be applied to fingers in order to observe any microbial reduction. In addition, a higher irradiation dose is to be applied to contaminated gloves, since, at least in some cases, gloves can also be disinfected, and it is assumed that they block far-UVC radiation. The latter is to be confirmed by optical transmission measurements.

## 2. Materials and Methods

For the bacterial reduction experiments, one of the authors irradiated the inner surface of his hands five times with and without gloves. In each case, both hands or gloves (models “nitrile white” from VWR (Radnor, PA, USA)) were first rubbed over the face and upper body to ensure a high concentration of microorganisms even on the sterile gloves. Subsequently, both hands were rubbed together to achieve an even distribution of microorganisms on both hands and gloves.

A filtered 222 nm far-UVC krypton-chloride excimer lamp from Ushio (Tokyo, Japan) was used as the radiation source. First, the distance from the lamp to the plane, where the irradiance was 1 mW/cm^2^, was determined with a photometer type X1-UV-3727 of Gigahertz-Optik (Tuerkenfeld, Germany). Then one contaminated hand was irradiated for 23 s to reach the dose of 23 mJ/cm^2^ (without gloves) and 100 s to reach the dose of 100 mJ/cm^2^ (with gloves). A maximum of one skin irradiation was performed per day to stay within the allowed limits in the EU. Three fingers of each of the irradiated and non-irradiated hands/gloves were pressed onto caso contact agar plates from VWR. The agar plates were then incubated for approximately 24 h at 37 °C. Afterwards, colonies were counted, and the ratio of colony numbers was determined.

For comparison, a similar disinfection study with contaminated gloves and fingers was performed with the commercial liquid alcohol-based hand disinfectant Sterilium of Bode Chemie (Hamburg, Germany). The application of the disinfectant was performed for 30 s according to WHO instructions [22], and with a following 2 min drying and waiting period before sampling.

In addition, the far-UVC transmission properties of some standard gloves were determined. First, pieces were first cut out of three different gloves. Among these glove models were the white nitrile gloves from VWR mentioned above, and also the “nitrile blue” models from Semperit (Vienna, Austria) and “latex white” from Braun (Schwalbach, Germany). Subsequently, the Gigahertz photometer was positioned in close proximity to the far-UVC lamp, and the irradiance on the detector was determined with and without a glove layer.

## 3. Results and Discussion

**Figure 1:**
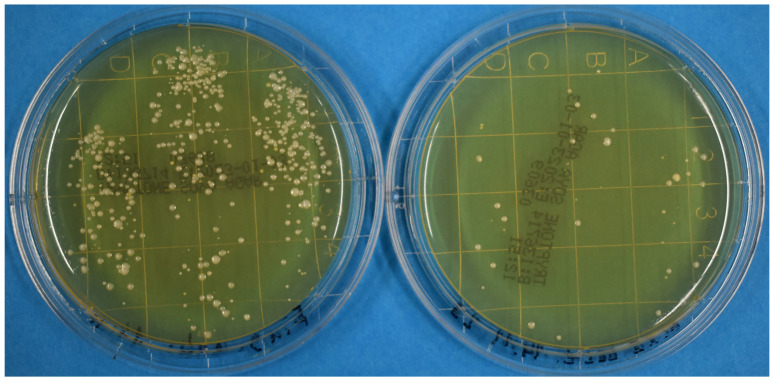
Agar plates after 24 h. On the left side, a plate from three unirradiated fingers; on the right, a plate from three fingers irradiated with 23 mJ/cm^2^.

**Figure 2:**
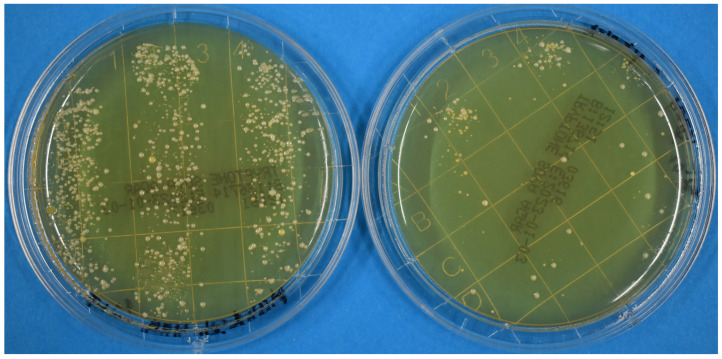
Agar plates after 24 h. On the left side, a plate from three unirradiated gloved fingers; on the right, a plate from three gloved fingers irradiated with 100 mJ/cm^2^.

Exemplary results of agar plates on unirradiated and irradiated hands and gloves after 24 h are presented on the right and left side in Figure 1 and Figure 2, respectively. A reduction in microorganisms was clearly visible for each individual experiment. The average number of counted colonies for unirradiated and irradiated fingers were 705 and 72 colonies, respectively. For nonirradiated and irradiated gloves, the corresponding values were 976 and 76 colonies, respectively. On average, the dose of 23 mJ/cm^2^ on naked skin reduced the number of colonies by approximately one log level (1.04 ± 0.13). For the dose of 100 mJ/cm^2^ on the gloves, a reduction of 1.33 ± 0.15 log levels was observed. Expressed as percentage values, the average reduction caused by the far-UVC irradiation was 88.2% (±4.3%) for the bare fingers and 93.6% (±2.5%) for the contaminated gloves.

Without gloves, the measured far-UVC irradiation directly in front of the lamp was 8.1 mW/cm^2^. The gloves reduced the measured far-UVC irradiation by a factor of 90,000 on average. (VWR nitrile white: 0.13 µW/cm^2^ or 1.6 × 10^−3^%, Semperit nitrile blue: 0.09 µW/cm^2^ or 1.1 × 10^−3^%, Braun latex white: 0.07 µW/cm^2^ or 0.9 × 10^−3^%).

These results prove that a considerable reduction in microorganisms can be achieved with the dose of about 23 mJ/cm^2^ currently allowed in the EU. However, compared to published results of irradiations in solutions, the inactivation success is rather low.

Surprisingly, the results on gloves were not very different from the results without gloves, although with 100 mJ/cm^2^, approximately four times the far-UVC dose was applied. It would have been expected that gloves have fewer folds than skin, and thus microorganisms are less protected from radiation.

Regarding the alcohol-based disinfectant, the average number of counted colonies for untreated and disinfected fingers were 447 and 4.6 colonies, respectively. For untreated and disinfected gloves, the corresponding values were 983 and 166 colonies, respectively. The alcohol-based hand disinfectant led to an average reduction of about two log levels (1.89 ± 0.10) on the bare skin and about one log level (0.91 ± 0.22) for the gloves. Expressed as percentage values, the average reduction caused by the alcohol-based disinfectant was 98.5% (±0.4%) for the bare fingers and 81.7% (±5.5%) for the contaminated gloves.

What are the consequences of these results for the possible application of far-UVC radiation for hand disinfection? With the current EU limits, multiple daily hand disinfection (without gloves) by far-UVC radiation would not even be permitted, although it could still be investigated whether reduced irradiation doses could possibly achieve similar reduction effects. However, should the new ACGIH limits also become valid in Europe—and if further studies on the effect of far-UVC radiation on skin confirm the apparent benign nature of this radiation—far-UVC use for hand disinfection would again be conceivable.

When gloves are used, there is initially no apparent risk of exceeding the existing radiation limits, even with more than 100 hand disinfections per day, because the glove material reduces the far-UVC radiation intensity by several orders of magnitude. An increase in the radiation dose would also be technically conceivable.

The danger here lies in possible glove perforations that are not immediately detected. Depending on the existing exposure limit value and the intended irradiation dose, the skin could be irradiated above the threshold dose even if gloves are used.

The far-UVC disinfection results reported here are not better or even worse than the results with the commercial hand disinfectant. However, there are two important aspects: (1) The available alcohol-based disinfectants are the result of decades of development and testing, and this is the first far-UVC application for hand disinfection, and it might be improved. Additionally, in contrast to alcohol-based disinfectants, far-UVC should even be able to inactivate bacterial spores, which was not investigated here. (2) According to many authors, the hand disinfection compliance of healthcare workers—usually performed with alcohol-based disinfectants—is lacking [1,2,3,23], with reasons including skin eczema, which makes the contact with alcohol very unpleasant, and a lack of time. A far-UVC-irradiation might be performed faster in the future and without this kind of skin irritation.

A future technical implementation of such a far-UVC radiation hand disinfection could consist of a box into which hands are inserted at the side and then irradiated from above and below. Cameras could be applied to monitor the correct execution of the disinfection process and even detect glove perforations.

## 4. Conclusions

Hand disinfection by means of far-UVC radiation is conceivable. In this study, a clear antimicrobial effect was observed both directly on the hand and on gloves. As a study limitation, it must be mentioned that the study presented here did not distinguish between different bacteria. Different bacteria probably exhibit different UV sensitivities. For less UV-sensitive bacteria, the applied irradiation doses could lead to a lower reduction. Further studies are desirable in this regard.

An actual future realization of far-UVC disinfection also depends on the assumed very low far-UVC-sensitivity of human skin or on irradiation limits resulting from it. This aspect has not been investigated here. Recent studies came to the conclusion that filtered far-UVC radiation is not a relevant hazard for skin; however, these studies are not very numerous, and the existing or discussed worldwide limits are very different.

## Data Availability

Not applicable.
